# Case Report: Elevated myocardial enzymes in a patient with metastatic rectal cancer without myocardial injury

**DOI:** 10.3389/fonc.2025.1681634

**Published:** 2026-01-12

**Authors:** Yalei Zhao, Xiaopeng Ma, Xiaojing Chang, Mingda Li, Yinghao Hao, Guojian Zhang, Zhesen Tian

**Affiliations:** 1 Department of Anus and Intestine Surgery, The Second Hospital of Hebei Medical University, Shijiazhuang, Hebei, China; 2 Department of Radiotherapy, The Second Hospital of Hebei Medical University, Shijiazhuang, Hebei, China; 3 School of Basic Medical Sciences, Hebei Medical University, Shijiazhuang, Hebei, China

**Keywords:** cardiac enzymes, case report, creatine kinase, laboratory tests, metastatic colorectal cancer

## Abstract

In clinical practice, myocardial injury is commonly screened using electrocardiograms and high-sensitivity troponin, but tumor patients may present with abnormalities in myocardial enzymes-a routine panel including creatine kinase (CK), CK-MB isoenzyme, lactate dehydrogenase (LDH), hydroxybutyrate dehydrogenase (HBDH), and myoglobin (MYO)-without corresponding ECG or troponin changes. This phenomenon may be related to tumor burden and tumor invasion rather than primary heart disease. Dynamic monitoring of myocardial enzyme abnormalities in patients with metastatic colorectal cancer has not been reported. This case provides a detailed analysis, offering clinical evidence for exploring the relationship between myocardial enzymes and tumor occurrence and development. A 67-year-old male patient with advanced rectal cancer and multiple metastases received chemotherapy with oxaliplatin and capecitabine, targeted therapy with bevacizumab, denosumab for bone metastases, and local radiotherapy. Dynamic monitoring during the treatment showed that the levels of myocardial enzymes significantly increased at the initial stage of metastasis, but there was no evidence of myocardial injury. When the tumor partially responded to the treatment, the expression levels of myocardial enzymes decreased. However, when the condition deteriorated and the number of metastatic lesions increased, the expression levels of myocardial enzymes sharply rose again, eventually accompanying the patient’s death. The non-cardiac injury-related increase in myocardial enzymes is closely associated with tumor burden and cancer activity, and can serve as a dynamic indicator for evaluating the efficacy of radiotherapy and chemotherapy and disease progression.

## 1 Introduction

Colorectal cancer ranks third among cancer-related causes of death (1). Early primary colorectal cancer can be removed endoscopically or surgically, but advanced tumors often metastasize and require multidisciplinary assessment and treatment, with a poor prognosis for patients (2, 3). The liver, lungs, distant lymph nodes and peritoneum are common sites of metastasis for colorectal cancer; while bone metastasis is relatively rare, if it occurs, it indicates a poor prognosis, with a median survival of less than 10 months and a 5-year survival rate of only 8.1% for patients (4–6). With the intensification of population aging, elderly cancer patients often have multiple system complications such as heart, brain and kidney diseases (7, 8). In clinical diagnosis and treatment, systematic assessment and examination are needed to avoid related risks.

Creatine kinase (CK) was historically used in myocardial infarction (MI) diagnosis, but current guidelines prioritize cardiac troponins and high-sensitivity cardiac troponins (hs-cTn) as the diagnosis biomarkers (9). CK now serves as an auxiliary marker to supplement hs-cTn results or monitor muscle injury severity. It is a dimer composed of M and B subunits. Currently, four isoenzymes have been identified: CK-MM, CK-MB, CK-BB, and mitochondrial CK (10). The first three are mainly distributed in the cytoplasm: CK-MM is mainly present in skeletal muscle and cardiac muscle; CK-MB is mainly located in cardiac muscle and has a lower content in skeletal muscle; CK-BB is mainly expressed in brain tissue, bladder, prostate, and gastrointestinal tract; while mitochondrial CK is distributed in mitochondria (11). When myocardial cells are damaged, the level of serum CK-MB rises rapidly, reflecting the release of a large amount of muscle enzymes into the blood. In patients with myocardial infarction, the proportion of CK-MB activity to total CK activity can increase from 6% to 25%, but it is usually less than 30% (11). However, in certain circumstances, such as malignant tumors, brain injuries, severe shock syndromes and hypothermia, CK levels can also rise significantly, which poses a challenge for clinical disease localization and differential diagnosis (11). For cancer patients, the changes in myocardial enzyme levels before and after radiotherapy and chemotherapy need to be carefully identified by clinicians as to whether they are pseudo-elevations caused by the tumor itself or myocardial damage resulting from radiotherapy and chemotherapy (12). This requires a comprehensive assessment of the patient’s cardiac function and tumor burden before and after treatment to adjust the treatment plan in a timely manner.

This case report presents a 67-year-old male patient with advanced rectal cancer who experienced multiple episodes of abnormal elevation of cardiac enzymes during the course of his illness. However, there was always a lack of direct evidence of myocardial injury (such as normal electrocardiogram and negative high-sensitivity troponin). The fluctuations in the patient’s cardiac enzyme levels were closely related to the activity of the cancer, significantly increasing during the initial stage of metastasis and progression, and significantly decreasing after radiotherapy and chemotherapy, suggesting that this abnormality may be due to non-myocardial injury mechanisms related to cancer (such as tumor release or inflammatory response), rather than primary heart disease. This case provides a clinical reference for the pseudo-elevation of cardiac enzymes in patients with metastatic cancer and emphasizes the need to be vigilant about the impact of tumor burden in the absence of cardiac symptoms.

## 2 Case report

A 67-year-old male visited the anorectal surgery department on March 7, 2025, due to bloody stools for one week. The patient experienced changes in bowel habits, with 4–5 bowel movements per day, accompanied by a sense of urgency and a feeling of rectal fullness. There was no abdominal pain, diarrhea, abdominal distension, nausea, or vomiting. The patient had no history of hypertension, diabetes, coronary heart disease, etc. Five years ago, he underwent an operation for varicose veins of the left lower extremity. Through digital rectal examination (in the knee-chest position), a cauliflower-like mass was palpable at 5 to 7 o’clock, 1 cm from the anal margin, and the index finger was stained with blood upon withdrawal.

**Figure 1:**
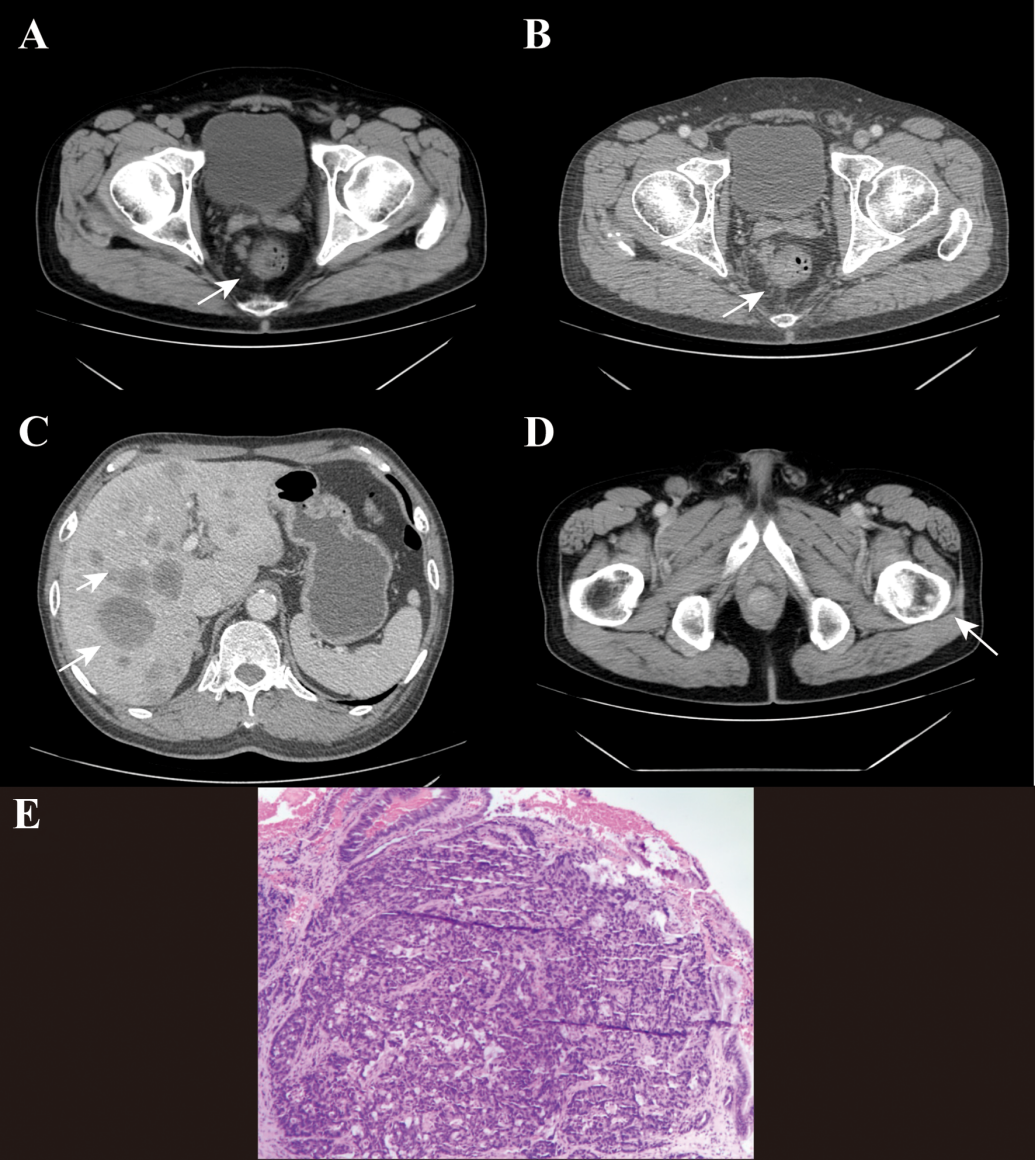
The plain and enhanced computed tomography (CT) scan images of the patient’s abdomen at admission and the pathological biopsy under colonoscopy of the patient. **(A)** Abdominal CT plain scan shows thickening of the intestinal wall at the terminal rectum. **(B)** Enhanced abdominal scan of the patient shows significant enhancement of the rectal wall, multiple adjacent lymph nodes and slightly increased density of the mesentery. **(C)** Multiple low-density, nearly round shadows with indistinct margins are visible in the liver. **(D)** A nodular slightly high-density shadow is visible on the left femur, with a long diameter of approximately 0.9 cm. **(E)** The patient’s pathological biopsy showed adenocarcinoma.

**Figure 2:**
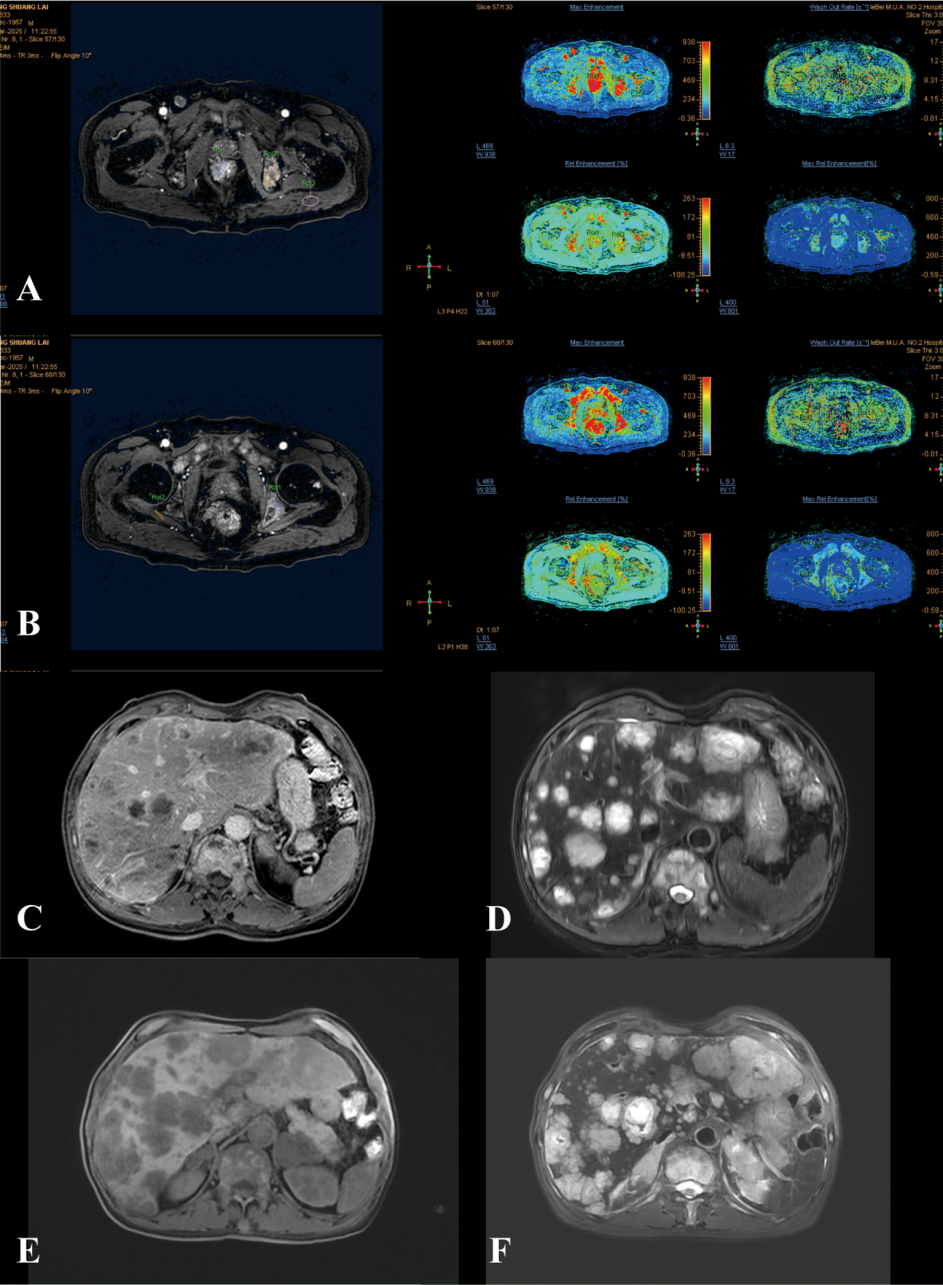
The changes in magnetic resonance (MR) during the patient’s treatment process. **(A, B)** The T1-weighted image of the rectum and the color T1 image of the metastatic lesion on MR at the time of hospital admission of the patient. **(C, D)** The T1 and T2 weighted magnetic resonance images of liver metastases during the stable period after radiotherapy and chemotherapy for the patient. **(E, F)** T1- and T2-weighted MR images of liver metastases in the terminal stage of the patient’s tumor.

The plain and enhanced abdominal CT scan showed the following (**Figures 1A, B**): The terminal rectal wall was thickened, with the thickest part approximately 1.5 cm, and it showed significant enhancement on the enhanced scan. Multiple lymph nodes were found nearby, with the largest one having a short diameter of about 1.3 cm. The mesentery density was slightly increased, and multiple lymph nodes were visible within the scanning range. The right inguinal lymph node was relatively large, with a short diameter of about 1.6 cm. Multiple low-density, round-like shadows were seen in the liver, with blurred edges. The largest one was located in segment II, measuring approximately 4.6×5.2 cm, and no significant enhancement was observed on the enhanced scan (**Figure 1C**). A nodular, slightly high-density shadow was visible in the left femur, with a long diameter of about 0.9 cm (**Figure 1D**). The plain and enhanced rectal magnetic resonance imaging (MR) (**Figures 2A, B**) showed that the lower edge of the tumor was less than 5 cm from the anal verge, the thickest part of the intestinal wall was 1.4 cm in the oblique transverse section, the tumor invaded the peritoneal fold, and there was metastasis in the rectal mesenteric fascia and inguinal lymph nodes; multiple metastases were found in the pelvis, femur and sacrum. Based on the rectal MR, the preliminary staging was T3N1M1. The patient was in the advanced stage of rectal cancer, so it was recommended to be transferred to the radiotherapy department for further treatment.

After being transferred to the radiotherapy department on the sixth day after admission, the patient underwent a PET/CT scan. The examination revealed thickening of the lower rectal wall with an irregular margin and heterogeneous hypermetabolism; multiple hypermetabolic soft tissue nodules and lymph nodes were found in the mesorectum, presacral region, right internal iliac vessels, right inguinal region, para-aortic region and porta hepatis. Multiple low-density round-like shadows were seen in the liver, accompanied by high or ring-shaped metabolism. Small nodules were scattered in both lungs, some of which were slightly hypermetabolic. A hypermetabolic soft tissue nodule was found in the left adrenal gland. Multiple hypermetabolic areas were observed throughout the bones. The diagnosis was rectal cancer with multiple metastases in the lymph nodes, liver, both lungs, left adrenal gland and bones (T3N2bM1).

**Figure 3:**
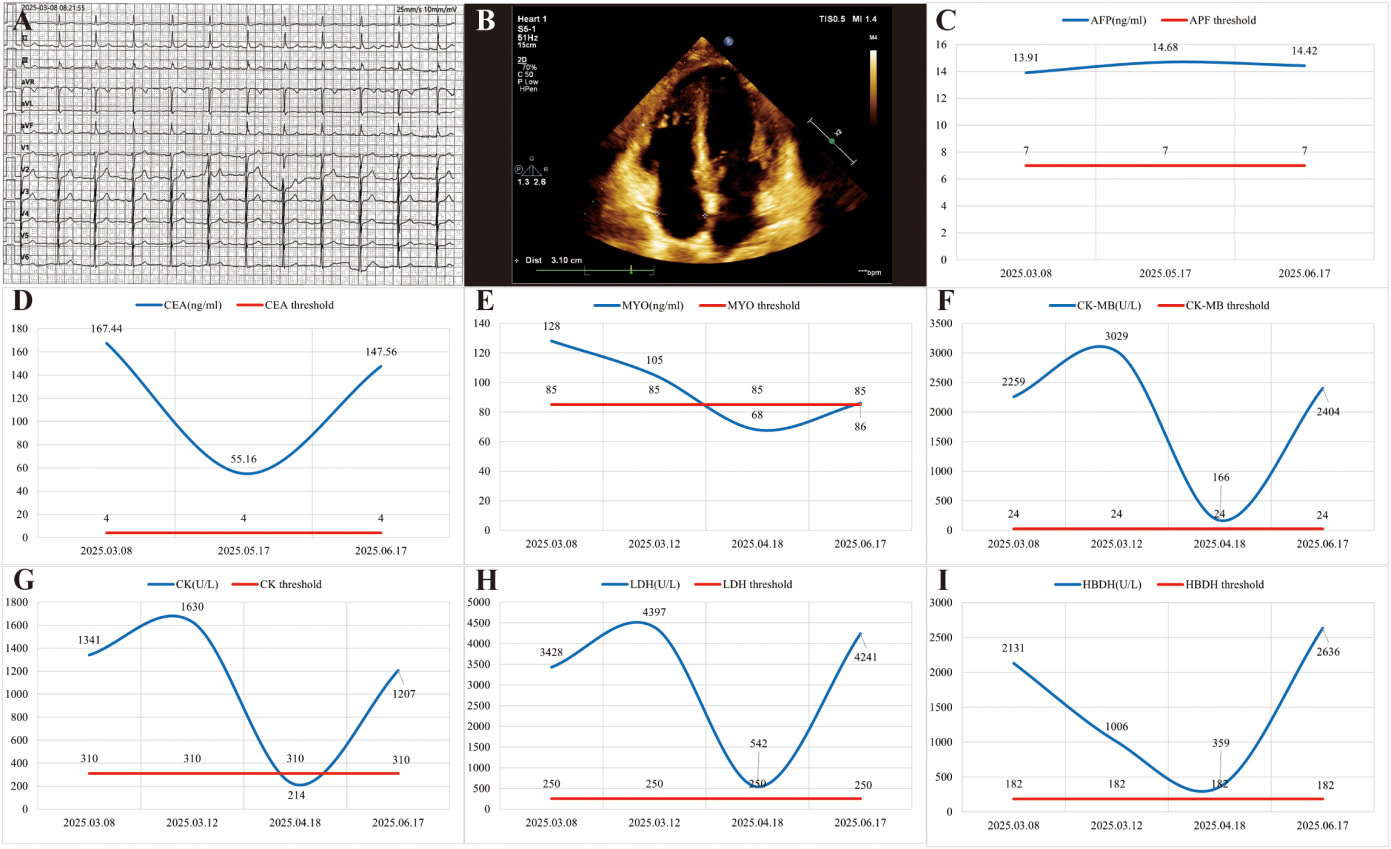
The patient presented with elevated myocardial enzymes without myocardial injury. **(A)** The electrocardiogram of the patient upon admission showed normal. **(B)** The echocardiogram performed upon the patient’s admission revealed normal findings. **(C)** The changes of alpha-fetoprotein (AFP) in patient during the diagnosis and treatment process. **(D)** The changes of carcinoembryonic antigen (CEA) in patient. **(E-I)** The change curve of five myocardial enzymes in patient during the diagnosis and treatment process. Figures E to I respectively show the changes in myoglobin (MYO), creatine kinase-MB (CK-MB), creatine kinase (CK), lactate dehydrogenase (LDH) and hydroxybutyrate dehydrogenase (HBDH).

The patient’s five myocardial enzymes (CK, CK-MB, LDH, MYO, HBDH) were persistently abnormal from the second day of hospitalization through to the sixth day, when they were transferred to the radiotherapy department. However, the patient’s electrocardiogram and echocardiogram showed no abnormal manifestations (**Figures 3A, B**). On the sixth day after admission, the test for high-sensitivity troponin in the patient also showed no abnormalities. It was initially suspected that the multiple metastases in the patient might have caused a false increase in myocardial enzymes or an increase in myocardial enzymes due to non-myocardial injury. A 24-hour electrocardiogram monitoring was conducted, and the results showed sinus rhythm with an average heart rate of 73 beats per minute, no pauses longer than 2.0 seconds, 254 atrial premature beats, accounting for less than 1% of the total heartbeats, and 215 ventricular premature beats, accounting for less than 1% of the total heartbeats.

After the biopsy of the rectum during colonoscopy, the pathology report indicated rectal adenocarcinoma (**Figure 1E**). The immunohistochemical staining results showed: MLH1(+), MSH2(+), MSH6(+), and PMS2(+). The patient’s microsatellite status was stable, and immunotherapy was not recommended at the moment. Therefore, the patient was treated with oxaliplatin and capecitabine chemotherapy combined with bevacizumab targeted therapy, and denosumab was used for bone metastasis. On the 12th day after admission, radiotherapy was initiated with the following plan: GTV was the lesion in the rectum, expanded by 0.5 cm to become PGTV; GTVnd was the enlarged lymph nodes in the groin and around the rectum, expanded by 0.5 cm to become PGTVnd; CTV was the lymphatic drainage area of the mesorectum. CTV1 was the lesion in the sacrum and ilium, expanded by 0.5 cm to become PTV1. DT: PGTVnd and PGTV were 55Gy/2.2Gy/25f, and PTV/PTV1 were 45Gy/1.8Gy/25f.

One month after radiotherapy, myocardial enzymes were retested and all indicators showed significant decreases, with myoglobin and creatine kinase returning to normal ranges. Two months after radiotherapy, the patient returned to our hospital for a follow-up examination and further treatment. The patient’s MR pelvic plain scan and enhanced scan showed that the local lesion in the rectum had improved compared to before (considered PR) (**Figures 2C, D**), so bevacizumab was suspended and oxaliplatin + capecitabine chemotherapy and denosumab for bone metastasis were administered.

Three months after radiotherapy, the patient underwent further re-examination and treatment. The patient complained of upper abdominal distension and pain. An upper abdominal MR scan revealed multiple metastases in the liver, bilateral adrenal glands, and thoracolumbar vertebrae, with an increase in lesions compared to the first hospitalization. A new abnormal enhancement nodule was observed in the tail of the pancreas, suggesting metastasis. Considering the overall condition, the disease was progressing (**Figures 2E, F**). The five-item myocardial enzyme test (CK, CK-MB, LDH, MYO, HBDH) showed abnormal elevations in all items, but a high-sensitivity troponin test was normal. It was suggested to change the chemotherapy drugs or conduct immunotherapy, but the patient refused the treatment plan and was treated with oxycodone for pain relief. The patient died in July during follow-up. **Figures 3C, D** show the changing trends of alpha-fetoprotein and carcinoembryonic antigen expressions in the patient’s tumor markers. **Figures 3E–I** show the levels of myocardial enzymes during the treatment period. The CARE checklist is in **Supplementary File 1**.

## 3 Discussion

**Figure 4:**
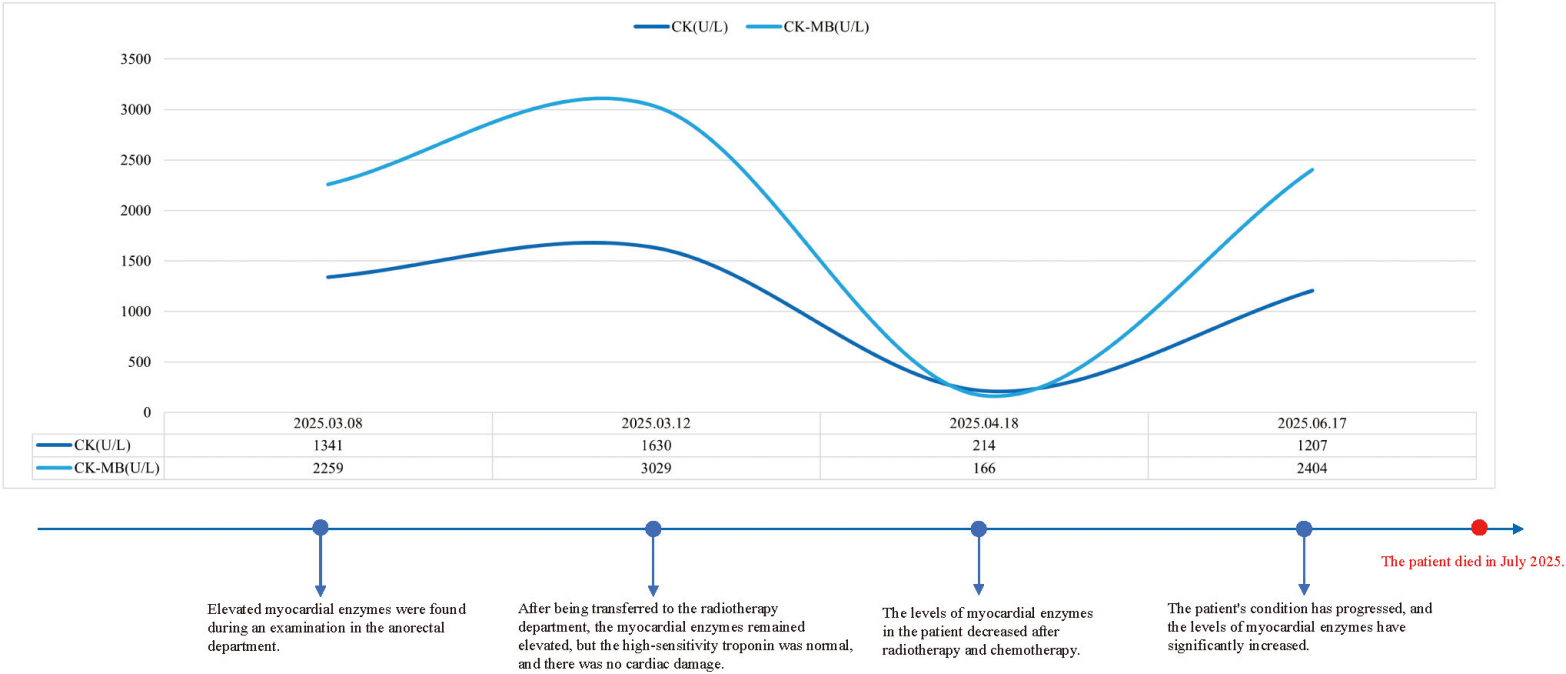
The changes in the patient’s myocardial enzymes and the timeline of treatment.

This patient was a 67-year-old male diagnosed with advanced metastatic rectal cancer (T3N2bM1). During the course of the disease, there were repeated abnormal elevations in the five cardiac enzymes, but the electrocardiogram, echocardiogram, and high-sensitivity troponin tests remained normal throughout, supporting that the elevation of cardiac enzymes was non-myocardial injury-related (possibly due to tumor burden or non-cardiac factors). After initial radiotherapy and chemotherapy intervention (including oxaliplatin + capecitabine combined with bevacizumab), the cardiac enzyme indicators significantly improved, with some returning to normal, demonstrating the therapeutic effect on non-cardiac factors. However, when the disease progressed and the number of metastases increased, the cardiac enzymes rose again, accompanied by the patient’s death, highlighting the close association between disease deterioration and the recurrence of abnormal cardiac enzymes. **Figure 4** shows the disease development timeline of this patient.

Due to the relatively poor sensitivity and specificity of CK and CK-MB, it is no longer recommended to use them as markers for diagnosing MI (9, 13). Elevated serum CK levels are not only seen in myocardial injury but also in various hereditary or acquired myopathies, such as muscular dystrophy, metabolic myopathy, congenital myopathy, drug/toxin-induced myopathy, inflammatory myopathy, and endocrine myopathy. Additionally, non-myopathy factors may also lead to elevated CK levels, including tumors, high fever, intense exercise, trauma, viral infections, malignant hyperthermia, and severe sunburn (14). Many studies have reported elevated CK and CK-MB levels in cancer patients. Chen et al. analyzed CK, CK-MB, and CK-MB/CK in 118 patients with pancreatic cancer, 72 patients with benign pancreatic masses, and 68 healthy controls. The results showed that the CK level in pancreatic cancer patients (55 IU/L) was lower than that in patients with benign masses (74.5 IU/L) and healthy controls (98.5 IU/L), which was different from previous reports. The researchers did not analyze the CK-MB and CK levels in pancreatic cancer patients at different stages, and further investigation is needed. However, the CK-MB/CK ratio in pancreatic cancer patients (0.260) was higher than that in patients with benign masses (0.173) and healthy controls (0.148), and the CK-MB/CK ratio was even higher in advanced pancreatic cancer patients (15). Chang et al. analyzed 846 patients with CK-MB/CK > 1.0 and found that the CK-MB/CK ratio in the malignant tumor group was significantly higher than that in the non-malignant tumor group (1.35 ± 0.28 vs 1.25 ± 0.23, p < 0.001). The most common malignant tumor with a CK-MB/CK ratio > 1.0 was colorectal cancer (1.42 ± 0.28, 16.5%) (16). B I Lee et al. reported a case of a lung cancer patient with elevated CK serum levels (17). Enzyme separation detection showed significant increases in CK-MB and CK-BB isoenzymes, but no myocardial injury or inflammation was found clinically or pathologically. Subsequent analysis of CK isoenzymes in tumor tissue showed that CK-MB and CK-BB were dominant, indicating that the increase in CK isoenzymes in circulation was not of myocardial origin but of tumor origin. Takayo Ota et al. reported a case of an 82-year-old male patient with elevated CK-MB but no myocardial injury. CK electrolyte analysis showed that the CK-MB range was normal, but the CK-BB level was elevated (18). Similarly, Abass Eidizadeh et al. reported a case of a 75-year-old patient with metastatic prostate cancer, with elevated total CK activity (387 U/L; normal value 30–200 U/L) and significantly elevated CK-MB activity (669 U/L; normal value ≤ 25 U/L). CK isoenzyme analysis by agarose gel electrophoresis revealed a band of giant CK-2 type (19). A study by Li et al. demonstrated that over 44% of patients with a CK-MB/CK ratio greater than 1.0 were diagnosed with malignant tumors. Moreover, the CK-MB/CK ratio was significantly higher in patients with malignant tumors compared to those without. Patients with secondary or metastatic tumors had significantly higher serum CK-MB activity, total CK activity, and CK-MB/CK ratio than those with primary tumors. Patients with multiple metastatic tumors had a significantly higher CK-MB/CK ratio than those with single metastatic tumors. Additionally, the CK-MB/CK ratio was significantly higher in patients with colorectal cancer and prostate cancer compared to other types of tumors. This suggests that the CK-MB/CK ratio could serve as a potential molecular marker for screening metastatic and malignant tumors. Abnormal creatine kinase in tumor patients is primarily caused by an increase in CK-BB content in the blood, leading to a false increase in CK-MB (11). CK-BB concentration is highest in normal brain tissue and only exists in small amounts in tissues such as the gastrointestinal tract, uterus, and vascular wall (20). Once these areas are invaded and damaged by tumors, CK-BB enters the blood, causing an increase in CK-MB detected by the immunosuppressive method. Secondly, abnormal CK appears in the blood, including giant CK1 and giant CK2, which are not inhibited by M antibodies, thus leading to a false increase in CK-MB activity (11).

In clinical practice, when tumor patients present with abnormal myocardial enzyme levels, priority should be given to investigating and ruling out myocardial injury-related diseases. However, reliance solely on laboratory test results is insufficient, as it is crucial to remain vigilant that tumor-associated factors causing myocardial enzyme abnormalities may obscure genuine myocardial damage. To avoid such misdiagnosis, a comprehensive evaluation should be conducted by integrating multiple myocardial-specific diagnostic modalities, including electrocardiography, echocardiography, and high-sensitivity troponin assays. If these diagnostic results are all negative, particular attention should be paid to the potential correlation between elevated myocardial enzymes (especially CK and its isoenzymes) and tumor burden. In such cases, the clinical focus should shift towards actively controlling tumor burden, while regularly monitoring changes in myocardial enzyme levels to dynamically assess tumor status and treatment efficacy.

It is a pity that in this case, we did not have time to conduct a subtype analysis of CK isoenzymes. However, in advanced tumor patients, the fluctuation of CK isoenzyme levels must have certain clinical patterns and significance. Current research cases and literature indicate that more clinical studies are needed in this field to explore the relationship between CK isoenzymes and specific tumors as well as their impact on tumor prognosis. Next, we will investigate whether the level of myocardial enzymes can be used as a monitoring indicator for the efficacy of radiotherapy and chemotherapy and the progression of patients’ tumors. In addition, exploring the impact of the tumor microenvironment on the release of creatine kinase may lay the foundation for the development of new biomarkers in the future.

## Data Availability

The original contributions presented in the study are included in the article/**Supplementary Material**. Further inquiries can be directed to the corresponding author.

## Supplementary material

Supplementary File 1 the CARE checklist.

## References

[1] Sung H Ferlay J Siegel RL Laversanne M Soerjomataram I Jemal A . Global cancer statistics 2020: GLOBOCAN estimates of incidence and mortality worldwide for 36 cancers in 185 countries. CA Cancer J Clin. (2021) 71:209–49. doi: 10.3322/caac.21660, PMID: 33538338

[2] Wang HH Yan YY Zeng HY Wang Y Ni KM Yu XR . The efficacy and safety of neoadjuvant and adjuvant chemo(radio)therapy combined with surgery in patients with locally advanced rectal cancer harboring defective mismatch repair system: a large-scale multicenter propensity score analysis. Front Immunol. (2025) 16:1626438. doi: 10.3389/fimmu.2025.1626438, PMID: 40692776 PMC12277327

[3] Silva GL de Moura EG Bernardo WM Leite de Castro V Morais C Baba ER . Endoscopic versus surgical resection for early colorectal cancer-a systematic review and meta-analysis. J Gastrointest Oncol. (2016) 7:326–35. doi: 10.21037/jgo.2015.10.02, PMID: 27284463 PMC4880782

[4] Shamohammadi M Garavand AA Mohammadtaheri B Alemrajabi M Mousavie SH Mehrjardi AZ . Solitary metastases of colon adenocarcinoma to the ankle: A case report and literature review. Int J Surg Case Rep. (2025) 133:111515. doi: 10.1016/j.ijscr.2025.111515, PMID: 40578238 PMC12246623

[5] Lahham EE Al-Sa'ed J Azzam M Warda A Al Amleh H . A case of solitary metastatic colon adenocarcinoma of the sternum: an unusual metastatic site. J Surg Case Rep. (2024) 2024:rjae656. doi: 10.1093/jscr/rjae656, PMID: 39421337 PMC11483571

[6] Assi R Mukherji D Haydar A Saroufim M Temraz S Shamseddine A . Metastatic colorectal cancer presenting with bone marrow metastasis: a case series and review of literature. J Gastrointest Oncol. (2016) 7:284–97. doi: 10.3978/j.issn.2078-6891.2015.092, PMID: 27034798 PMC4783735

[7] Brunello A Sandri R Extermann M . Multidimensional geriatric evaluation for older cancer patients as a clinical and research tool. Cancer Treat Rev. (2009) 35:487–92. doi: 10.1016/j.ctrv.2009.04.005, PMID: 19481353

[8] Reddy P Shenoy C Blaes AH . Cardio-oncology in the older adult. J Geriatr Oncol. (2017) 8:308–14. doi: 10.1016/j.jgo.2017.04.001, PMID: 28499724 PMC5776715

[9] Thygesen K Alpert JS Jaffe AS Chaitman BR Bax JJ Morrow DA . Fourth universal definition of myocardial infarction (2018). Circulation. (2018) 138:e618–618e651. doi: 10.1161/CIR.0000000000000617, PMID: 30571511

[10] Aydin S Ugur K Aydin S Sahin İ Yardim M . Biomarkers in acute myocardial infarction: current perspectives. Vasc Health Risk Manage. (2019) 15:1–10. doi: 10.2147/VHRM.S166157, PMID: 30697054 PMC6340361

[11] Li Y Chen Y Shao B Liu J Hu R Zhao F . Evaluation of creatine kinase (CK)-MB to total CK ratio as a diagnostic biomarker for primary tumors and metastasis screening. Pract Lab Med. (2023) 37:e00336. doi: 10.1016/j.plabm.2023.e00336, PMID: 37767053 PMC10520525

[12] Shil S Kumar P Mumbrekar KD . Cancer therapy-induced cardiotoxicity: mechanisms and mitigations. Heart Fail Rev. (2025) 30:1075–92. doi: 10.1007/s10741-025-10531-0, PMID: 40481994 PMC12297282

[13] Byrne RA Rossello X Coughlan JJ Barbato E Berry C Chieffo A . 2023 ESC Guidelines for the management of acute coronary syndromes. Eur Heart J. (2023) 44:3720–826. doi: 10.1093/eurheartj/ehad191, PMID: 37622654

[14] Rong P Zhao S Fu Q Chen M Yang L Song Y . Case report: One child with an autism spectrum disorder who had chronically elevated serum levels of CK and CK-MB. Front Psychiatry. (2022) 13:995237. doi: 10.3389/fpsyt.2022.995237, PMID: 36147964 PMC9485572

[15] Chen C Lin X Lin R Huang H Lu F . A high serum creatine kinase (CK)-MB-to-total-CK ratio in patients with pancreatic cancer: a novel application of a traditional marker in predicting Malignancy of pancreatic masses. World J Surg Oncol. (2023) 21:13. doi: 10.1186/s12957-023-02903-3, PMID: 36653771 PMC9847085

[16] Chang CC Liou CB Su MJ Lee YC Liang CT Ho JL . Creatine kinase (CK)-MB-to-total-CK ratio: a laboratory indicator for primary cancer screening. Asian Pac J Cancer Prev. (2015) 16:6599–603. doi: 10.7314/apjcp.2015.16.15.6599, PMID: 26434881

[17] Lee BI Bach PM Horton JD Hickey TM Davis WA . Elevated CK-MB and CK-BB in serum and tumor homogenate of a patient with lung cancer. Clin Cardiol. (1985) 8:233–6. doi: 10.1002/clc.4960080409, PMID: 2985311

[18] Ota T Hasegawa Y Murata E Tanaka N Fukuoka M . False-positive elevation of CK-MB levels with chest pain in lung adenocarcinoma. Case Rep Oncol. (2020) 13:100–4. doi: 10.1159/000505724, PMID: 32231530 PMC7098329

[19] Eidizadeh A von Ahsen N Friedewald S Binder L . Macro-CK type 2 in metastatic prostate cancer. Diagnosis (Berl). (2019) 6:307–9. doi: 10.1515/dx-2018-0039, PMID: 30412465

[20] Forouzan A Fahimi MA Bastan A Delirrooyfard A . Diagnostic competence of creatine kinase BB, in mild traumatic brain injury and its prognostic value. Adv BioMed Res. (2023) 12:84. doi: 10.4103/abr.abr_122_21, PMID: 37200752 PMC10186058

